# Cell therapy in kidney diseases: advancing treatments for renal regeneration

**DOI:** 10.3389/fcell.2024.1505601

**Published:** 2024-12-11

**Authors:** Amankeldi A. Salybekov, Aiman Kinzhebay, Shuzo Kobayashi

**Affiliations:** ^1^ Qazaq Institute of Innovative Medicine, Regenerative Medicine Division, Cell and Gene Therapy Department, Astana, Kazakhstan; ^2^ Kidney Diseases and Transplant Center, Shonan Kamakura General Hospital, Kamakura, Japan

**Keywords:** cell therapy, chronic kidney diseases, acute kidney injury, kidney diseases, mesenchimal stem cells, CD34 cells

## Abstract

Kidney diseases, including acute kidney injury (AKI) and chronic kidney disease (CKD), pose a significant global health challenge, with high morbidity and mortality rates driven by rising prevalence of risk factors such as diabetes and hypertension. Current therapeutic strategies are often limited, prompting the exploration of advanced cell therapies as potential solutions. This review provides a comprehensive overview of the state of cell therapies in kidney disease, tracing the progression from preclinical studies to clinical applications. Recent studies highlited that cell-based interventions offer kidney-protective properties through mechanisms such as paracrine signaling, immune modulation, and direct tissue integration, demonstrating potential in both AKI and CKD settings. Despite promising results, challenges remain in optimizing cell therapy protocols, including cell sourcing, delivery methods, and long-term outcomes. Finally, the review addresses on efforts to enhance cell function, optimize dosing, and refine delivery techniques to improve clinical outcomes in kidney disease management.

## 1 Introduction

Kidney diseases, including acute kidney injury (AKI) and chronic kidney disease (CKD), represent a significant global health burden. According to recent statistics, CKD affects approximately 9%–16% of the global population, while AKI impacts around 13.3 million individuals annually (Liu et al., 2022; Stevens et al., 2023; Hill et al., 2015; Mehta et al., 2015). These conditions are associated with high morbidity and mortality rates, and their management remains a major challenge due to the limitations of current therapeutic strategies and the growing prevalence of underlying risk factors such as diabetes and hypertension (Jha et al., 2013; Kovesdy et al., 2022).

In recent years, advanced cell therapies have emerged as promising interventions in the treatment of kidney diseases. These innovative approaches aim to restore kidney function and mitigate disease progression by leveraging various cell-based products, including stem cells and progenitor cells. The transition from preclinical studies to clinical application has been marked by significant advancements, highlighting the potential of these therapies to revolutionize kidney disease management (Brosnahan et al., 2021; Morizane et al., 2022; Rota et al., 2019; Wong, 2021). Preclinical research has demonstrated the efficacy of cell transplantation in animal models of kidney disease, showing improvements in kidney function, tissue regeneration, and reduction in fibrosis (Papazova et al., 2015; Liu et al., 2020; Rizvi et al., 2021; Wang et al., 2023). These findings have paved the way for clinical trials, where the focus has been on translating these promising results into human applications. Recent updates in clinical trials have showcased the safety and efficacy of various cell-based interventions, although challenges remain in optimizing protocols and addressing issues related to cell sourcing, delivery methods, and long-term outcomes (Lee et al., 2023; Wong, 2021; Rota et al., 2019; Zhang et al., 2020).

This review provides a comprehensive overview of the current state of advanced cell therapies in kidney disease treatment, tracing the journey from preclinical research to clinical application. It aims to highlight the latest developments in cell transplantation and the ongoing efforts to refine and translate these therapies into effective clinical solutions. By synthesizing recent findings and clinical updates, this review offers insights into the future directions of cell-based therapies and their potential impact on kidney disease management.

## 2 Cell therapy in kidney diseases: lessons learned from preclinical studies

Cell therapy has demonstrated therapeutic potential for a range of acute and chronic conditions, including kidney diseases. Recent years have seen a marked increase in preclinical studies involving cell therapy in animal models, with some cell products advancing to clinical settings. This section discusses insights gained from preclinical studies and their potential clinical applications.

Induced pluripotent stem cells, commonly abbreviated as iPS cells, represent a form of stem cells with the capability to develop into multiple cell types. They are created directly from ordinary somatic cells. Preclinical studies have revealed that induced pluripotent stem cells (iPSCs) possess kidney-protective properties due to their paracrine effects (Collino et al., 2020). Extracellular vesicles (EVs) derived from iPSCs help protect kidneys by mitigating cell death, tissue damage, and inflammation. These EVs preserve mitochondrial integrity by maintaining mitochondrial mass and preventing oxidative damage, and regulate critical genes involved in oxidative stress defense, such as *SOD1*, *SOD3*, *TXN1*, *TXNRD2*, and *GSTK1*. Additionally, iPSC-EVs carry molecules such as CD326 (EpCAM) and CD133 (prominin-1), which are crucial for renal regeneration and immune modulation (Collino et al., 2020). iPSC-EVs have shown greater efficacy in these protective roles compared to adipose-derived mesenchymal stem cell (MSC)-EVs. However, further research is needed to fully understand their composition and safety for clinical use. While iPSCs offer advantages over MSCs, such as prolonged survival, a homogeneous cell population, and high expansion rates, they are also associated with higher costs, approximately $800,000 for clinical-grade lines, which are not always covered by health insurance (Huang et al., 2019). MSCs are multipotent progenitor cells of mesodermal origin, which are able to self-renew and differentiate into different specialized cell lineages of skeletal tissues, such as: chondrocytes (cartilage cells), osteoblasts (bone cells) and adipocytes (fat cells) (Nombela-Arrieta et al., 2011). MSC therapy for kidney diseases is more extensively documented in scientific literature compared to other cell types (Huang and Yang, 2021). This is largely due to the availability of these cells, established protocols for isolation and culture, and their allogeneic transplant potential. This potential arises from the absence of HLA-II molecules, which are encoded in the human major histocompatibility complex (MHC) by three main gene families: HLA-DR, HLA-DP, and HLA-DQ, making MSCs preferable over other cell types. In another study, Heer et al. were the first to demonstrate that adult-derived Spermatogonial Stem Cells (SSCs), originating from germ stem cells in the seminiferous epithelium of the testis, can initiate early renal differentiation (Heer et al., 2013). SSCs, when co-cultured, were observed to differentiate into renal-specific phenotypes, suggesting possible paracrine mechanisms. *In vivo* studies showed that GFP-labeled SSCs integrated into host kidney tissue, forming nephron-like structures. Both stromal and epithelial cells derived from SSCs were identified, indicating multi-lineage differentiation potential. Although SSC-derived cells exhibited lower levels of differentiation markers compared to native kidney cells, this research establishes a foundation for further investigation into the clinical potential of SSCs in nephrogenesis (Heer et al., 2013).

Another study demonstrated that murine germline progenitor stem cells (GPSCs) could differentiate into renal tubular-like cells *in vitro* and effectively restore kidney function *in vivo* following AKI. After 6 days in culture, GPSCs began expressing mesodermal genes, with some differentiating into cells expressing kidney-specific proteins such as KSP. When injected into mice with induced renal ischemia-reperfusion injury (IRI), these differentiated cells migrated to the kidneys, engrafted long-term, and resulted in a significant increase in Y chromosome-positive cells 6 weeks post-injury. The treatment also led to upregulation of heme oxygenase-1 (*H O -1*), an enzyme that protects against oxidative stress, suggesting a mechanism for renal protection. Importantly, differentiated GPSCs, as opposed to undifferentiated ones, were crucial in mitigating renal damage and preventing fibrosis, tubular dilation, and other CKD hallmarks. This research supports the potential of GPSCs in renal repair, underscoring their plasticity and ability to prevent CKD progression (De Chiara et al., 2014).

Endothelial colony-forming cells (ECFCs) originate from progenitor cells found in circulating peripheral blood. A position paper from the International Society on Thrombosis and Haemostasis highlights research that has identified a unique molecular and phenotypic profile for ECFCs. This profile is characterized by the presence of specific endothelial markers, such as CD31, CD144, CD146, epidermal growth factor-like domain 7 (EGFL-7), and vascular endothelial growth factor receptor 2 (VEGFR2), along with the absence of hematopoietic markers like CD45 and CD14 (Smadja et al., 2019). Burger et al. (2015) were the first to investigate the effects of human cord blood-derived Endothelial Colony Forming Cells (ECFCs) and their EVs in IRI-induced AKI. Their findings indicated that ECFCs and their exosomes, but not microparticles (MPs) or other vesicle-depleted media, effectively protect renal endothelial cells from apoptosis. This protection is associated with reduced plasma creatinine levels, tubular necrosis, and inflammation. Unlike ECFCs, MPs may exacerbate endothelial injury. The study suggests that ECFC-derived exosomes could be a targeted therapeutic approach for AKI, offering benefits such as lower immunogenicity and reduced risk of tumor formation compared to whole-cell therapies. However, further research is required to elucidate the specific mechanisms of these protective effects and to refine therapeutic strategies (Burger et al., 2015). Later Patschan et al. explored the therapeutic effects of ECFCs in experimental IRI-induced AKI (Patschan et al., 2017). They found that a single dose of ECFCs administered post-ischemia improved kidney function, reduced fibrosis, and decreased markers of Endothelial-to-Mesenchymal Transition (EndoMT) and endothelial acetylated α-tubulin in some cases. However, ECFCs did not prevent loss of peritubular capillary density (PTCD) nor significantly modulate the autophagic flux marker p62. Early Endothelial Progenitor Cells (eEPCs) proved more effective in preserving endothelial integrity and reducing EndoMT. The study suggests that ECFCs’ protective effects may be mediated through indirect mechanisms, such as exosome release, rather than direct endothelial incorporation. Differences in efficacy between eEPCs and ECFCs are attributed to their distinct mechanisms of action, particularly the role of the secretome. Future research should explore alternative administration protocols and further investigate the secretome’s role in therapeutic efficacy. While ECFCs can reduce fibrosis and temporarily improve kidney function, they are less effective than eEPCs, particularly in preserving PTCD, which is critical for preventing CKD progression. Understanding these differences may enhance the therapeutic use of ECFCs and eEPCs in AKI management (Patschan et al., 2017).

Recent studies have also highlighted the potential of regeneration-associated cells (RACs) and their extracellular vesicles (RACevs) as novel regenerative sources for AKI treatment (Salybekov al., 2024; Ohtake et al., 2018). Evidence shows that a small number of RACs or RACevs can restore kidney function even in severe ischemic injury models (Salybekov al., 2024; Ohtake et al., 2018). The regenerative paracrine activity of transplanted RACs is potentially linked to mechanisms that mitigate tissue damage and improve renal function. It has been demonstrated that RACev secretes more EVs than BMMSCs and carry beneficial anti-inflammatory, anti-fibrosis, anti-apoptosis, and angiogenesis-associated miR (Salybekov et al., 2021). As previously mentioned, RACs contribute to vasculogenic (characterized by an increased presence of CD34+/CD133+ cells) and anti-inflammatory (marked by CD206+ cells and regulatory T cells) environments within the target tissues (Salybekov et al., 2018; Salybekov et al., 2019). Additionally, it has been observed that a small subset of RACs express CD31 markers. Consequently, it can be inferred that a portion of the transplanted RACs interacts with local endothelial cells to restore peritubular capillaries that are damaged during the recovery phase of the AKI model.

Sum up, Despite the promising results from various preclinical trials, translating these findings to clinical settings has faced challenges. Issues include the failure of promising animal study results to replicate in larger animal or human trials, potential biases in animal models, and the inability of rodent models to fully represent human disease states. The procedures for cell isolation, cell culture, and harvesting, as well as the method and dosage for transplantation, lack standardization in animal studies. This clarity is essential for effectively translating preclinical research into clinical practice. Additionally, rodent models used for cell therapy are often young compared to the middle-aged or elderly human population affected by CKD and AKI. Furthermore, kidney diseases in humans are frequently accompanied by other conditions, such as cardiovascular or gastrointestinal diseases, which also impact kidney recovery.

## 3 Acute kidney injury

AKI is a significant global health concern, impacting approximately 13.3 million individuals annually, with incidence rates on the rise (Mehta et al., 2015). AKI is defined by a rapid increase in serum creatinine levels by ≥ 0.3 mg/dL (≥26.5 μmol/L) within 48 h, or a rise to ≥1.5 times the baseline within the previous 7 days. It may also present with a decrease in urine output to ≤0.5 mL/kg/h for 6 h, or a combination of these factors (Khwaja, 2012). This prevalent clinical syndrome can arise from a range of causes, including renal ischemia, sepsis, toxic effects of drugs, and injuries related to myoglobin or hemoglobin. The progression from AKI to CKD and end-stage renal disease (ESRD) is well-documented, with many patients ultimately requiring renal replacement therapy (RRT) or transplantation (Fu et al., 2023). The likelihood of developing CKD and ESRD correlates with the severity of AKI. A notable meta-analysis revealed that patients with a normal glomerular filtration rate (GFR) prior to AKI had a higher relative risk of advancing to ESRD compared to those who developed AKI with a pre-existing reduced baseline GFR (Su et al., 2022). Specifically, the absolute risk of ESRD was 9.8% for patients with a reduced baseline GFR without AKI, which increased to 18.4% upon AKI onset. Conversely, those with normal baseline GFR had an absolute risk of 0.4% without AKI, rising to 4.6% with AKI, representing a more than tenfold increase (Wald et al., 2009). Although patients with normal GFR who develop AKI face a lower absolute risk of ESRD, their relative risk is elevated due to the initially low probability of ESRD (Wald et al., 2009). The presence of pre-existing proteinuria further influences the risk of CKD development following AKI. While recovery from AKI can mitigate the risk of progressing to ESRD, persistent subclinical damage may contribute to adverse outcomes despite AKI often being reversible based on serum creatinine levels (Coca et al., 2012).

The pathogenesis of AKI is frequently associated with sepsis, ischemia-reperfusion injury, and immune-mediated injury post-kidney transplantation. Sepsis-induced AKI is driven by hyper-inflammatory responses from infections involving pathogens, damage-associated molecular patterns, inflammatory cytokines, and vasoactive agents (Dellepiane et al., 2017). These responses lead to endothelial and tubular epithelial cell damage, cytokine storms, and microvascular complications (Cantaluppi et al., 2014). IRI exacerbates AKI through hypoxia-induced damage and oxidative stress during reperfusion, resulting in vasoconstriction, inflammation, and immune cell infiltration, which causes cell detachment and tissue damage in the kidneys (Dellepiane et al., 2017; Bonventre and Yang, 2011). In kidney transplantation, immune-mediated injury can significantly impact graft health. T-cell-mediated rejection involves CD8^+^ T-lymphocytes that induce apoptosis and necrosis of tubular epithelial cells (TECs), similar to changes observed in ischemic and nephrotoxic AKI (Dellepiane et al., 2017; Einecke et al., 2010). Antibody-mediated rejection involves donor-specific antibodies targeting allo-HLA antigens and other molecules, resulting in graft damage through complement activation or non-complement fixing mechanisms (Dellepiane et al., 2017).

Current research into cell-based therapies for AKI includes the use of MSCs, iPSCs, spermatogonial stem cells (SSCs), proangiogenic cells (PACs), and ECFCs (Table 1). MSCs are actively employed in both completed and ongoing clinical trials, while the other cell types have been primarily studied in preclinical settings. SSCs and ECFCs have shown direct repair mechanisms, whereas iPSCs, MSCs, and PACs primarily exert their effects through indirect mechanisms, such as paracrine signaling and microvesicle-dependent processes (Patschan et al., 2018; Dellepiane et al., 2017). The following sections will delve into clinical research involving MSCs and relevant preclinical studies with other mentioned cell types.

**TABLE 1:** Clinical cell therapy trials in AKI.

Study title	Conditions	Interventions	Cell dose	Primary outcomes	Locations	Study status	NCT number
A Study of Cell Therapy for Subjects With Acute Kidney Injury Who Are Receiving Continuous Renal Replacement Therapy	Acute Kidney Injury	SBI-101, MSC	Low: 250 × 10^6^ cells; High: 750 × 10^6^ cells	Safety and tolerability as measured by incidence of IP-related serious adverse events	Allentown, Pennsylvania, United States	Unknown	NCT03015623
Clinical Trial of Mesenchymal Stem Cells in the Treatment of Severe Acute Kidney Injury	Acute Kidney Injury	MSC	10^6^ cells/kg	eGFR	Beijing, China	Unknown	NCT04194671
A Study of Cell Therapy in COVID-19 Subjects With Acute Kidney Injury Who Are Receiving Renal Replacement Therapy	COVID-19|Acute Kidney Injury|Sepsis	SBI-101, MSC	2.5 × 10^8^/cells, 7.5 × 108/cells	Safety and tolerability as measured by incidence of IP-related serious adverse events	United States	Unknown	NCT04445220
Allogeneic Multipotent Stromal Cell Treatment for Acute Kidney Injury Following Cardiac Surgery	Kidney Tubular Necrosis, Acute	Multipotent Stromal Cells, MSC	1 × 10^6^/cells/kg	Absence of MSC-specific Adverse or Serious Adverse Events	Utah, United States	Completed	NCT00733876
A Study to Evaluate the Safety and Efficacy of AC607 for the Treatment of Kidney Injury in Cardiac Surgery Subjects (ACT-AKI)	Acute Kidney Injury	AC607	2 × 10E6 hMSC/kg body weight	Time to Kidney Recovery defined as a post-operative serum creatinine return to pre-operative baseline values	United States, Canada	Terminated	NCT01602328

### 3.1 CD34^+^ cell therapy for acute kidney injury

Suzuki et al. recently documented the first human case in a phase I/II clinical trial involving the transplantation of autologous granulocyte colony-stimulating factor-mobilized peripheral blood CD34-positive cells for the treatment of severe acute kidney injury (Suzuki et al., 2021). The CD34^+^ cells were administered directly into each renal artery using a syringe driver, delivering 45 million CD34-positive cells in 50 mL saline per artery at a rate of 150 mL/h, totaling 90 million cells. At 23 weeks post-therapy, the patient’s serum creatinine levels decreased to 2.96 mg/dL (Suzuki et al., 2021). The study observed that the estimated glomerular filtration rate, averaging 0.11 mL/min/1.73 m^2^/day from day 15 to day 44 after the withdrawal of hemodialysis, improved to 0.25 mL/min/1.73 m^2^/day from day 45 to day 73 post-transplantation, suggesting a potential role of cell transplantation in the recovery of kidney injury (Suzuki et al., 2021). It is important to note that this report is based on a single case, and further studies with larger sample sizes are necessary to determine the efficacy of CD34^+^ cells in patients with AKI.

### 3.2 Mesenchymal stem cell therapy for acute kidney injury

MSCs have emerged as promising therapeutic agents for kidney diseases due to their capacity for multidirectional differentiation, migration, homing, and paracrine signaling (Lee et al., 2021). Preclinical studies have demonstrated that MSCs can be both effective and safe in the treatment of organ injuries (Wang et al., 2013; Brasile et al., 2019). MSC therapy may facilitate renal function recovery in kidney disease through mechanisms including anti-inflammatory effects, anti-apoptotic actions, angiogenesis promotion, oxidative stress reduction, fibrosis prevention, and modulation of autophagy and cellular senescence (Chen et al., 2023).

**FIGURE 1:**
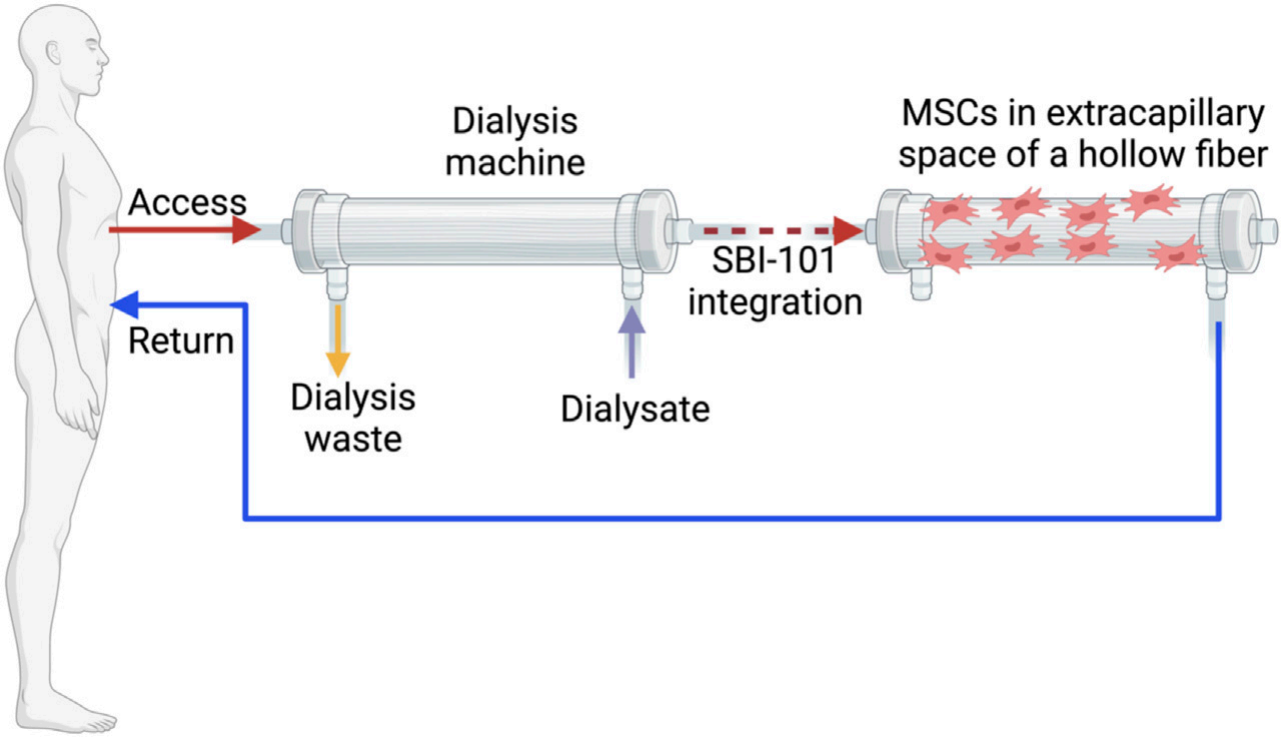
An extracorporeal circuit equipped with the integrated SBI-101 system. Created in BioRender

However, early-phase trials conducted in 2018 (NCT01602328, Table 1) indicated potential limitations of traditional MSC therapy as a treatment for AKI (Swaminathan et al., 2018). This phase II trial, based on promising preclinical rat AKI model studies (Monsel et al., 2014) as well as a phase I clinical trial, (Gooch et al., 2008) revealed that allogeneic MSCs (AC607) did not significantly improve recovery times for kidney function compared to placebo in patients undergoing cardiac surgery. Secondary outcomes also showed no significant differences, with trends numerically favoring placebo. Although not statistically significant, a higher incidence of dialysis requirement or mortality was observed in patients treated with AC607, and recovery of kidney function was prolonged in both primary and sensitivity analyses (Swaminathan et al., 2018). These findings suggest that MSC therapy may be more effective for preventing AKI rather than treating it once it has developed. The intricate clinical context of AKI related to cardiac surgery, including extended cardiopulmonary bypass times and ongoing renal injury, may obscure any modest clinical benefits of MSCs. Additionally, reduced renal blood flow during surgery and pre-existing kidney impairments could limit MSC effectiveness. There is also a potential for adverse effects or harm from MSCs in certain inflammatory conditions. Despite high cell viability, the study did not confirm the precise delivery and distribution of MSCs to the kidneys. It is possible that the extent of clinical injury might exceed the repair capabilities of both endogenous and exogenous MSCs. Thus, further investigation into the assumptions regarding the adequacy and viability of administered MSCs is necessary to assess their applicability and impact on outcomes (Swaminathan et al., 2018). Subsequent studies have suggested that the issues observed with MSC therapy might not be inherent to the MSCs themselves, but rather related to the methodologies used. Swaminathan et al. have demonstrated the anti-inflammatory effects of MSCs through their paracrine action, marked by indicators such as IL-10 and TGF-β1 using SBI-101 system (Swaminathan et al., 2021). They developed SBI-101 system for MSC culturing inside of hemodialisis hollow fiber and released paracrine factors returned to the patient (Figure 1). Improved delivery routes and preconditioning strategies may enhance MSC functionality which were discussed in chapter 8 and 9.

## 4 AKI to CKD transition

The risk of AKI progression to CKD is notably high if intervention is not implemented to halt further deterioration. A longitudinal study spanning a decade has demonstrated that episodes of AKI can result in persistent renal dysfunction, with many patients failing to fully regain their baseline renal function (Ponte et al., 2008). Variables such as age, pre-existing comorbidities, and eGFR at discharge are significant predictors of long-term outcomes, while the severity and duration of AKI have a relatively minor impact. This underscores the necessity of viewing AKI not merely as an acute event but as a potential precursor to CKD, thus requiring diligent long-term monitoring and management (Ponte et al., 2008). A meta-analysis corroborates this by revealing similarly elevated risks of transitioning from AKI to CKD in both perioperative and non-perioperative settings, with odds ratios of 5.20 and 3.32, respectively (Abdala et al., 2021). For individuals who have suffered AKI, the incidence of developing CKD is 25.8 cases per 100 person-years, and progression to ESRD occurs at a rate of 8.6 cases per 100 person-years. Furthermore, patients with AKI are 8.8 times more likely to develop CKD, 3.1 times more likely to progress to ESRD, and twice as likely to die compared to those without AKI (Abdala et al., 2021). The pathophysiology underlying the transition from AKI to CKD involves several interconnected processes (Guzzi et al., 2019), which can potentially be modulated through cell therapy. Endothelial dysfunction, characterized by impaired blood flow due to vasoconstriction and vascular congestion, includes capillary rarefaction, decreased VEGF production, tissue hypoxia, inflammation, and fibrosis. Inflammation, driven by the infiltration of various immune cells including monocytes, macrophages, neutrophils, and T- and B-cells, impacts tissue damage and repair. Macrophages play a dual role, either promoting injury and inflammation through M1 polarization or aiding healing via M2 polarization. The activation of pathways such as IL-22, RA, and Wnt/β-catenin can either facilitate healing or exacerbate fibrosis and CKD progression. Fibrosis, marked by myofibroblast recruitment and extracellular matrix deposition, often follows pericyte detachment, capillary rarefaction, and tubular injury. While fibrosis initially serves as a protective mechanism by isolating damaged areas, chronic or recurrent fibrosis can exacerbate kidney damage. Recent findings have shown that extracellular vesicles derived from RACev effectively regulate post-AKI fibrosis, inflammation, and hypoxia, and promote enhanced angiogenesis (Salybekov et al., 2024). Notably, transplanted RACev preferentially accumulate in ischemia-injured proximal tubular cells, which are particularly vulnerable to ischemic damage. The RACev actively drive inflammation and epithelial-to-mesenchymal transition through the beneficial delivery of miRNAs (Salybekov et al., 2024).

In summary, AKI is often inadequately addressed by clinicians and researchers, and currently lacks specific treatments, with most cases being managed symptomatically. As a result, many patients who experience AKI eventually develop CKD, which underscores the need for early intervention through cell or EV therapy to prevent disease progression.

## 5 Chronic kidney diseases

CKD affects approximately 10% of the global adult population, resulting in 1.2 million deaths and 28 million years of life lost annually (GBD Chronic Kidney Disease Collaboration, 2020; Xie et al., 2018). By 2040, CKD is anticipated to become the fifth leading cause of death worldwide (Kalantar-Zadeh et al., 2021). CKD is a chronic condition characterized by progressive structural and functional alterations in the kidneys due to a range of etiologies. The disease’s progression involves key mechanisms including inflammatory responses, fibrosis, oxidative stress, and reactive oxygen species (ROS) production. Diagnosis is determined by a decrease in kidney function, reflected by an estimated glomerular filtration rate (eGFR) below 60 mL/min/1.73 m^2^ or the presence of kidney damage indicators such as albuminuria, hematuria, or imaging abnormalities that persist for at least 3 months. CKD is associated with significant morbidity and mortality and can advance to ESRD (Kalantar-Zadeh et al., 2021).

Diabetes mellitus is the leading global cause of CKD, followed by glomerulonephritis, hypertension, chronic pyelonephritis, adult polycystic kidney disease, renovascular disease, and other unspecified causes. As described in the following sections on clinical trials, the majority of studies focused on stem cell treatments for CKD are conducted at stages 3 and 4. The primary aim of these therapies is to halt further declines in GFR that could lead to ESRD. At stage 5, where complete kidney failure and extensive fibrosis render regeneration to a functional state unfeasible, kidney replacement through transplantation becomes necessary.

### 5.1 Autologous CD34^+^ cells therapy for chronic kidney disease

The therapeutic efficacy of the CD34^+^ cells, as a treatment for CKD is under investigation. Previous preclinical research has shown that therapy with EPCs can help preserve residual renal function in CKD models. This benefit is attributed to enhanced angiogenesis, improved blood flow, and reductions in oxidative stress, inflammation, and fibrosis (Huang et al., 2015). Building on these findings, a Phase I clinical trial was conducted to assess the efficacy of autologous CD34^+^ cells in patients with stage 3 and 4 CKD (Lee et al., 2017). Encouraging results from this initial trial informed the design of a subsequent Phase II clinical trial (Yang et al., 2020). Notably, this trial demonstrated that the administration of autologous circulatory-derived CD34^+^ cells in CKD patients was safe, with no serious adverse events (SAEs) reported. Additionally, the study tested the hypothesis that CD34^+^ cell therapy could preserve renal function and improve clinical outcomes in CKD patients.

The primary finding revealed a significant reduction in the incidence of dialysis or death at 1 year among patients receiving CD34^+^ cell therapy compared to those who did not, suggesting benefits beyond renal improvement. However, no significant changes were observed in creatinine clearance and circulatory creatinine levels after 12 months, indicating no additional short-term renal benefits. Adverse events, such as dialysis or mortality, were concentrated within the first 6 months, potentially due to the patients’ severe baseline conditions. The study has several limitations, including a small sample size that restricts the ability to draw definitive conclusions about efficacy and clinical outcomes, as well as a short follow-up period of 12 months without extensive long-term data. These factors may impact the statistical significance of the findings. Furthermore, the absence of a double-blind design introduces some uncertainty regarding the specific effects of G-CSF mobilized CD34^+^ cells (Yang et al., 2020).

Taking together, clinical trials investigating CD34-positive cell therapy in patients with CKD have shown controversial outcomes. To accurately assess the efficacy of CD34^+^ cells, larger randomized controlled trials with adequate sample sizes, strict inclusion and exclusion criteria, are needed. Additionally, personalized approaches to cell therapy may improve the therapeutic results.

### 5.2 Mesenchymal stem cell therapy for chronic kidney disease

In a study by Makhlough et al., observations regarding the use of bone marrow-derived MSC (BMMSCs) for CKD treatment were consistent with those for AKI. The primary aim of their Phase I trial was to evaluate the safety of this therapy, which was confirmed. However, secondary outcomes did not show a significant difference in eGFR post-treatment (Makhlough et al., 2018). This suggests that conventional intravenous administration of MSCs might present similar challenges in CKD treatment as observed in AKI. As previously noted, several factors contribute to these challenges, including decreased cell delivery to the target organ, accumulation in other internal organs, and the progression of CKD may be influenced by various complicated factors, including immunological, metabolic, and hemodynamic elements.

One potential solution for CKD patients is the use of stromal vascular fraction (SVF). SVF is a cellular mixture obtained from adipose tissue through liposuction, followed by collagenase treatment and washing of the lipoaspirate. The advantages of SVF include a higher yield of adipose-derived MSCs (ADMSCs)—approximately 500 times greater than from bone marrow—ease of access from the patient’s own fat tissue, which minimizes immune complications, and a rapid processing time of about 3 hours, thus reducing the time and cost associated with cell culture (Carstens et al., 2023). ELIXCYTE, a drug derived from this method, was administered to 12 CKD patients in three different cell doses: low, medium, and high. The Phase I clinical trial (NCT02933827, Table 2) demonstrated that the treatment was generally well-tolerated, with no significant difference in eGFR changes among the dose cohorts and only one instance of dose-limiting toxicity observed in the high-dose group. Although there were no significant differences in eGFR changes between doses, some patients did show improvement. The study suggests potential benefits from multiple doses and indicates better outcomes in patients with a baseline eGFR ≥30 mL/min. ADSCs are believed to facilitate endogenous repair and immunomodulation. Further research is needed to confirm these findings and to determine optimal dosing and frequency. Another clinical study (NCT05154591, Table 2) explores the use of SVF cells administered via renal artery catheterization to enhance kidney function in patients with chronic kidney disease of unknown cause (CKDu), previously known as Mesoamerican nephropathy (Yang et al., 2020). It predominantly affects young, non-diabetic, normotensive individuals who are frequently asymptomatic. By the time of diagnosis, most patients are already in CKD stages 3–4, indicating a rapid decline in kidney function (Carstens et al., 2023). The study assesses the safety and feasibility of using SVF cells via renal artery catheterization, suggesting that SVF may improve kidney function, especially in the early stages of the disease. The pathology of CKDu involves an acute kidney insult that progresses to chronic damage, highlighting the need for anti-inflammatory and pro-angiogenic interventions. Preliminary results indicate potential benefits of SVF, aligning with findings from studies on atherosclerotic renal disease treated with adipose MSCs, which show decreased inflammation, fibrosis, and improved vascular flow. Over a 36-month period, ultrasound studies showed a trend toward increased kidney volume following SVF treatment. At 12 months, 26 out of 36 kidneys were larger than their baseline size, and by 36 months, 34 out of 36 kidneys (excluding three patients who had died) remained larger. The mean renal resistive index (RRI) decreased from 0.68 at baseline to 0.55 at 36 months, indicating improvement. The study noted limitations such as a small sample size and lack of randomized controls. Future research will focus on early-stage disease and standardized dosing (Carstens et al., 2023).

**TABLE 2:** Clinical cell therapy trials in CKD.

Study title	Conditions	Cell dose	Outcomes	Locations	Study status	NCT number
Bone Marrow-Derived Mesenchymal Stem Cell Therapy for Chronic Kidney Disease	Chronic Kidney Diseases	Dose Arm 1: two IV infusions of 100 × 10^6^ allogeneic ADMSCs Dose Arm 2: single IV infusion of 200 × 10^6^ cells	Adverse events and/or SAE; Change in eGFR Value	Mayo Clinics, United States	Active Not Recruiting	NCT05362786
Stem Cell Therapy for Patients with Focal Segmental Glomerulosclerosis	Focal Segmental Glomerulosclerosis Chronic Kidney Diseases	N/A Bone marrow stem cell	Kidney injury (increase of serum creatinine); CKD (doubling of serum creatinine, dialysis); Potential differentiation disorders of transplanted cells; Systemic inflammatory potential of mononuclear cells administration in renal circulation; Death	Rio de Janeiro, Brazil	Completed	NCT02693366
Safety and Efficacy of Autologous Bone Marrow Stem Cells for Treating Chronic Renal Failure	Chronic Renal Failure	N/A Autologous bone marrow stem cells	Significant clinical improvement in serum creatinine and urine output (improvement in measured GFR by 50%); Number of patients reporting adverse effects as a measure of safety and tolerability	Karnataka, India	Unknown	NCT01152411
SVF (Adipose Tissue Derived MSC) Based Therapy for CKD.	Chronic Kidney Diseases	Low: 5 mL (5–10) *10^5^ cells; Medium: 5 mL (10–20) *10^5^ cells High: 5 mL (>20) *10^5^ cells SVF Containing Autologous Non-Expanded ADSC	Incidence of MAEs, SAEs; Change from baseline to 24 weeks visit in GFR and split renal function in all patients; Change from baseline to 24 weeks visit in eGFR with serum creatinine level in patients with CKD 4 and below; Change from baseline to 24 weeks visit in need for dialysis in patients with CKD 5	Dhaka, Bangladesh	Recruiting	NCT03939741
Safety of Cultured Allogeneic Adult Umbilical Cord Derived Mesenchymal Stem Cell Intravenous Infusion for CKD	Chronic Kidney Diseases	100*10^6^ cells AlloRx	Safety (adverse events)	St. John’s, Antigua and Barbuda	Recruiting	NCT05018845
Safety and Efficacy of KDSTEM Inj. in Patients with Chronic Kidney Disease	Chronic Kidney Diseases	Low: 1.0 × 10^8^ cells High: 3.0 × 10^8^ cells KDSTEM Inj. Urine derived cells	The number of subjects with treatment-related adverse events as assessed by CTCAE version 5.0	Daejeon, Chungcheongnam-do, Republic of Korea	Recruiting	NCT06071143
Safety and Efficacy of BMMNC in Patients with Chronic Renal Failure	Chronic Renal Failure	N/A IV transfer of Autologous BMMNCs	Maintain or demonstrate improvement in laboratory values	Pune, Maharashtra, India	Unknown	NCT01876017
Stem Cell Therapy for Chronic Kidney Disease	Chronic Kidney Diseases Diabetes Mellitus, Type 2 Diabetes Mellitus, Type 1 Diabetes Mellitus|Diabetic Nephropathies	Dose Arm 1: 2 IV 75 × 10^6^ cells at day 0 and month 3. Dose Arm 2: 1 IV 150 × 10^6^ cells at day 0 Allogeneic ADMSC	AEs and/or SAEs	Mayo Clinics, United States	Active Not Recruiting	NCT04869761
CKDu Treated with Intra-arterial Infusion of Autologous SVF Cells	Chronic Renal Failure of Unknown Cause	31.7*10^6^–142.7*10^6^ SVF cells Adipose-derived stromal vascular fraction cells	Incidence of treatment related AEs; Preliminary evidence of efficacy	Leon, Nicaragua	Completed	NCT05154591
Treatment of Chronic Renal Failure with Adipose Tissue-derived Mesenchymal Stem Cells	MSCs Chronic Kidney Diseases Renal Interstitial Fibrosis	N/A	Serum creatinine	Jiangsu, China	Unknown	NCT03321942
Clinical Trial to Evaluate Safety of Allogeneic Bone Marrow Derived Mesenchymal Stem Cell in Chronic Kidney Disease	Chronic Kidney Disease Stage 3B|Chronic Kidney Disease stage4	Cellgram-CKD 10 mL	Incidence of AE and the level of the AE analyzed according to the Common Terminology Criteria for Adverse Event (CTCAE) (version 5.0)	Seoul, Republic of Korea	Completed	NCT05042206
Rituximab Combined with MSCs in the Treatment of PNS (3–4 Stage of CKD)	Renal Insufficiency, Chronic Nephrotic Syndrome	10^6^/kg/time every 2 weeks MSC	The change of serum creatinine elevation; The percentage of ESRD or death	Guangzhou, Guangdong, China	Unknown	NCT02966717
MIC Cell Therapy for Individualized Immunosuppression in Living Donor Kidney Transplant Recipients	Kidney Failure, Chronic	N/A Mitomycin C-induced peripheral blood mononuclear cells (MICs)	Frequency of adverse events after intravenous administration of MICs within 30 days after transplantation	Heidelberg, Germany	Completed	NCT02560220
Stem Cells for Uremic Calciphylaxis Patients	Chronic Kidney Diseases Calciphylaxis Calcific Uremic Arteriolopathy Treatment	N/A Human amniotic mesenchymal stem cells	Wound Healing (The Bates-Jensen Wound Assessment)	Nanjing, Jiangsu, China	Active Not Recruiting	NCT04592640
Hypoxia and Inflammatory Injury in Human Renovascular Hypertension	Renal Artery Stenosis Ischemic Nephropathy Renovascular Disease Chronic Kidney Disease	N/A MSC	Change in Kidney function (Renal Tissue oxygenation); Safety of Mesenchymal stem cell infusion	United States	Completed	NCT02266394
Adipose-derived Stem Cells (ADSCs) for Moderate to Severe Chronic Kidney Disease	Moderate to Severe Chronic Kidney Disease	Low: 8 mL (6.4*10^7^ cells); Middle: 24 mL (19.2*10^7^ cells); High: 40 mL (32.0*10^7^ cells) ELIXCYTE (SVF)	Incidence of AEs and SAEs; Change from baseline to Week 24 visit in eGFR	Taiwan	Completed	NCT02933827

### 5.3 Renal autologous cell therapy

Renal autologous cell therapy (REACT) represents a promising new approach under investigation. Traditional cell-based therapies for CKD often involve intravascular administration of mesenchymal cells, which can vary in dosage, potency, stability, and efficacy. REACT seeks to address these issues with a personalized approach, using autologous, homologous cells to restore nephron structure, enhance renal function, and reduce CKD-related comorbidities. Specifically, REACT is being studied for its effects on advanced stages of diabetes-related CKD.

**FIGURE 2:**
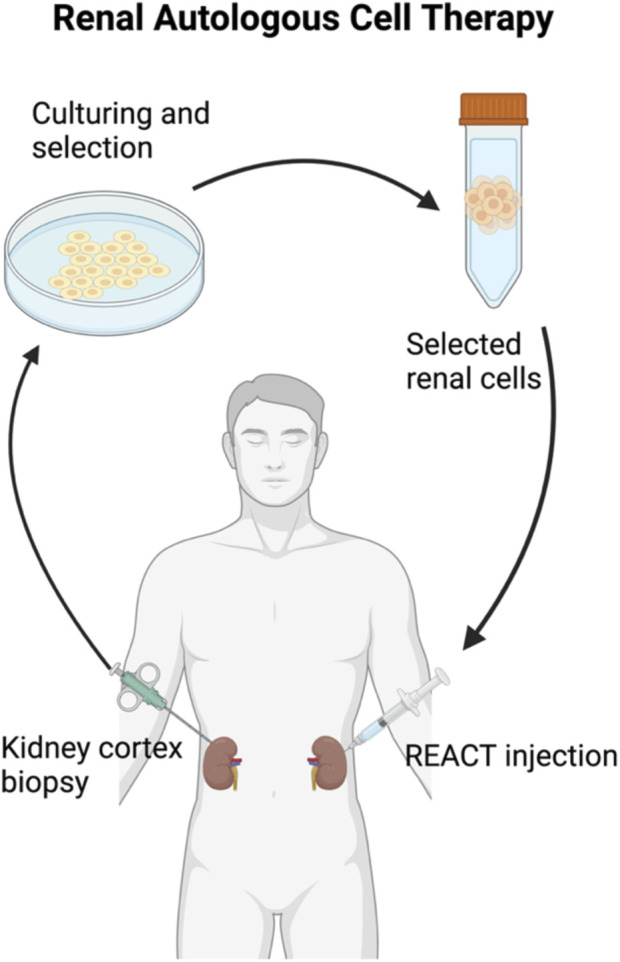
Renal autologus cell therapy. Created in BioRender

This method involves selected renal cells (SRCs) that are isolated and expanded in accordance with Good Manufacturing Practice (GMP) from kidney biopsy tissue of CKD patients (Figure 2). The SRCs used in REACT are believed to support kidney repair by activating developmental pathways similar to those observed during embryonic kidney formation. This hypothesis is reinforced by the presence of specific cellular markers in SRCs associated with nephrogenesis, such as SIX2, Osr1, and RET (Stavas et al., 2022). In a completed study (NCT02836574, Table 3) (Stavas et al., 2022), which involved patients with moderate to severe type 2 diabetic CKD (D-CKD), REACT therapy showed promising results. The treatment resulted in a statistically significant reduction in the decline of eGFR, indicating stabilization of kidney function. Notably, this improvement was achieved with two injections, differing from the single injection used in a previous phase I trial. The study identified three types of responses based on eGFR changes: high responders, moderate responders, and low responders, each demonstrating varying degrees of improvement or stabilization in renal function. Higher initial eGFR appeared to correlate with better outcomes, though the sample size was small. Additionally, the study suggested that SRCs might contribute to the formation of neo-kidney-like tissue, potentially leading to the observed improvements in renal function. Further research, including larger phase III trials, is needed to confirm these findings and determine the patient populations most likely to benefit from this therapy (Stavas et al., 2022). Rilparencel (NCT05018416, Table 3) is an investigational autologous cell therapy derived from a participant’s kidney cortex tissue, predominantly consisting of proximal tubular epithelial cells, aimed at renal repair. This ongoing study involves patients at CKD stages 3a–4 and either type 1 or type 2 diabetes. The study compares renal function outcomes between two groups: one receiving two doses (one per kidney) 3 months apart, and the other receiving a second dose only if there is a decline in eGFR or an increase in the urine albumin-to-creatinine ratio (UACR) (Stavas et al., 2024). Another ongoing study (NCT04115345, Table 3) of REACT therapy (Stavas et al., 2021) involves preliminary data from five patients with congenital anomalies of the kidney and urinary tract (CAKUT) related CKD. Initial results suggest that the study is feasible and safe for further clinical trials. The study uses percutaneous, image-guided techniques with conscious sedation, demonstrating the ability to administer REACT safely in patients with an eGFR <30 mL/min. Advantages of autologous kidney cell therapy include effective cell expansion, minimal immune complications, and precise delivery into the renal cortex. Potential complications, such as radiation exposure and bleeding, are minimized through dose-reduction strategies, careful anticoagulation assessment, and atraumatic needles. No transfusions were required, and sedation was well tolerated. REACT cells are well accepted by patients, with no immunologic or cell transformation issues. These cells can be expanded and preserved for future use, aiding in the repair of nephron structure and function. Initial results indicate improved renal function and stabilization of CKD in CAKUT patients (Stavas et al., 2021).

**TABLE 3:** Renal autologous cells (REACT) therapy for CKD.

Study title	Conditions	Cell dose	Outcomes	Locations	Study status	NCT number
A Study of Participants With Chronic Kidney Disease Previously Treated With REACT	Diabetic Kidney Disease|CAKUT|Chronic Kidney Diseases	N/A	1. Biopsy Related SAEs 2. Injection Procedure Related SAEs 3. Investigational Product Related SAEs 4. Treatment-Emergent SAEs	Boise, Idaho, United States	Recruiting	NCT05694169
A Study of Renal Autologous Cell Therapy (REACT) in Participants With Type 2 Diabetes and Chronic Kidney Disease	Type 2 Diabetes Mellitus|Chronic Kidney Diseases	N/A	The time from first injection to the earliest of: • At least 40% reduction in eGFR sustained for 30 days • eGFR <15 mL/min/1.73 m^2^ sustained for 30 days and/or chronic dialysis, and/or renal transplant • Renal or cardiovascular death	Spain	Not Yet Recruiting	NCT05286853
A Study of REACT in Subjects With Type 2 Diabetes Mellitus and Chronic Kidney Disease	Type 2 Diabetes Mellitus|Chronic Kidney Diseases	N/A	The time from first injection to the earliest of: At least 40% reduction in eGFR sustained for 30 days or eGFR <15 mL/min/1.73 m^2^ sustained for 30 days and/or chronic dialysis, and/or renal transplant or Renal or cardiovascular death	United States, Australia, Canada, Taiwan, United Kingdom	Recruiting	NCT05099770
A Study of Autologous Renal Autologous Cell Therapy (REACT) in Patients With Diabetic Chronic Kidney Disease	Type 2 Diabetes Mellitus|Chronic Kidney Disease	3 × 10^6^ cells/g estimated kidney weight	Incidence (percentage of subjects) with procedure and/or product related adverse events by System Order Class and Preferred Term	United States	Completed	NCT03270956
A Study of a Renal Autologous Cell Therapy (REACT) in Patients With Chronic Kidney Disease (CKD) From Congenital Anomalies of the Kidney and Urinary Tract (CAKUT)	Chronic Kidney Disease|Congenital Anomalies of Kidney and Urinary Tract	thermolabile gelatin-based hydrogel concentration of 100 × 10^6^ cells/mL	Assess change in eGFR and observe incidence of renal-specific procedure and/or product related AEs through 18 months following two Renal Autologous Cell Therapy (REACT) injections [Safety]	United States	Completed	NCT04115345
Study of Renal Autologous Cell Therapy (REACT) in Subjects With Type 1 or 2 Diabetes and Chronic Kidney Disease (REGEN-007)	Chronic Kidney Diseases|Type 1 Diabetes Mellitus|Type 2 Diabetes Mellitus	3×10^6^ cells/g estimated kidney weight; the dosing volume is 3.0 mL for each 100 g of kidney weight	Improvement in renal function progression rate: the change from pre-injection baseline value in total (acute + chronic) slope of eGFR. Procedural and investigational product-related treatment-emergent AEs (TEAEs) obtained through 18 months after the last REACT injection	United States	Active Not Recruiting	NCT05018416
A Study of Renal Autologous Cell Therapy (REACT) in Type 2 Diabetics With Chronic Kidney Disease	Type 2 Diabetes Mellitus|Chronic Kidney Disease	3 × 10^6^ cells/g estimated kidney weight	Change in Renal Function	United States	Completed	NCT02836574

## 6 Cell therapy in kidney transplantation immune modulation

The application of tailored immunosuppressive medications has significantly enhanced graft and patient survival during the early stages post-transplant. However, long-term outcomes remain suboptimal due to chronic graft rejection, which can ultimately result in transplant failure. Furthermore, prolonged immunosuppression is associated with severe side effects that adversely affect patient survival and quality of life, increasing susceptibility to infections. Numerous reports and strategies have explored the peri-transplant administration of hematopoietic and non-hematopoietic immunomodulatory cells, demonstrating their ability to induce tolerance safely and effectively in pre-clinical models of solid organ transplantation. Many preclinical findings have been successfully translated into clinical trials, yielding promising results Table 4. In the following sections, we will review the effectiveness of regulatory T cells and MSCs in kidney transplantation and immune tolerance.

### 6.1 Regulatory T Cells

Regulatory T cells (Tregs) typically express surface markers CD25^+^CD127-FoxP3+. Research into Treg biology has revealed that Treg dysfunction can lead to various autoimmune diseases, highlighting their crucial role in maintaining the balance between inflammatory and anti-inflammatory cell subsets. Additionally, Tregs secrete several paracrine factors that are beneficial. Infusion of autologous Tregs might reduce the need for immunosuppression in kidney transplant recipients. Recent studies by Roemhild et al. evaluated phase I/IIa Treg cell therapy in kidney transplant patients to compare the immunosuppressive effects of Tregs versus traditional immunosuppressive drugs. Their findings indicated that both the Treg-treated and control groups achieved 100% three-year allograft survival with comparable clinical and safety profiles. Notably, 73% of patients receiving Tregs achieved stable monotherapy immunosuppression, whereas the control group continued standard dual or triple drug regimens (Roemhild et al., 2020). Mechanistically, Treg administration reduced the activation of conventional T cells and shifted Tregs *in vivo* from a polyclonal to an oligoclonal T cell receptor repertoire (Roemhild et al., 2020). Sawitzki et al. summarized results from six parallel cell therapy group (CTG) trials included in the ONE Study, comparing polyclonal Tregs, donor-antigen reactive Tregs, tolerogenic autologous dendritic cells, and regulatory macrophages to a reference group receiving identical background immunosuppressive treatments (Sawitzki et al., 2020). Immunophenotyping results from these trials showed a significant reduction in Treg-specific demethylated region demethylation in the reference group but not in the CTG trials. Additionally, the reference group exhibited a significant increase in CD8^+^ TEMRA cells and CD8^+^CD57^+^ chronically activated T cells, while the CTG group showed more CD8^+^CD28^+^ T cells. Notably, regulatory cell therapy also resulted in higher anti-cytomegalovirus T-cell responses compared to the reference group (Sawitzki et al., 2020). This increase is likely related to inflammation-induced cytomegalovirus reactivation, observed only in the reference group (Manuel and Avery, 2021). Furthermore, CTG trial patients displayed increased mRNA expression of genes associated with immunosuppression-free operational tolerance (e.g., *MS4A1*) and co-inhibitory molecules (*CD200*), while showing reduced expression of rejection-associated genes (*HMMR*). These findings suggest a restoration of immune cell composition similar to healthy controls. Regulatory cell therapy has proven feasible and safe in living-donor kidney transplant recipients, associated with fewer infectious complications but similar rejection rates in the first year compared to the reference group (Sawitzki et al., 2020). Thus, immune cell therapy represents a potentially valuable approach to minimizing the burden of general immunosuppression and its complications in kidney transplant recipients.

**TABLE 4:** Cell therapy for solid organ transplant immune modulation.

Study title	Target diseases	Cell type	Cell dose	Locations	Status	Clinical trials identifier
Reference Group Trial for The ONE Study: A Unified Approach to Evaluating Cellular Immunotherapy in Solid Organ Transplantation	End-stage Renal Failure Kidney Graft Rejection	Regulatory M macrophages	Dose Arm 1 Transplantation of MSCs in two intravenous infusions of 100 × 10^6^ cells at time zero and 3 months Dose Arm 2 Transplantation of MSCs single dose 200 × 10^6^	Regensburg, Germany	Completed	NCT01656135
Umbilical Cord Mesenchymal Stem Cells Therapy for Diabetic Nephropathy	Diabetic Nephropathy	Human umbilical cord mesenchymal stem cells	Transplantation of 1 × 10^6^/kg MSCs once a 0, 4, and 8 weeks	Kunming, China	Recruiting	NCT04125329
Patient-Derived Stem Cell Therapy for Diabetic Kidney Disease	Diabetic Kidney Disease Diabetes Mellitus, Type 1 and 2 ESRD	Autologous adipose-derived Mesenchymal stem/stromal cells	Lower dose: two MSC infusions of 2.5 × 10^5^ cells/kg at time zero and 3 months; single kidney, intra-arterial delivery Higher dose Two MSC infusions of 5.0 × 10^5^ cells/kg at time zero and 3 months; single kidney, intra-arterial delivery	Mayo clinics, USA	Recruiting	NCT03840343
Stem Cell Therapy for Patients with Focal Segmental Glomerulosclerosis (STEFOG)	Focal Segmental Glomerulosclerosis Chronic Kidney Diseases	Bone Marrow derived Mononuclear stem cells	BMMSC at 30 to 100 million doses dissolved in plasma and divided injected into two renal arteries	Rio de Janeiro, Brazil	Completed	NCT02693366

### 6.2 Mesenchymal Stromal Cells and MSC-EVs

The immunomodulatory mechanisms underlying MSCs have been well established, and their efficacy has been confirmed in clinical trials for treating graft-versus-host disease (GVHD) (Kilian et al., 2021). Due to their strong immunosuppressive effects, MSCs have garnered attention for use as induction therapy in kidney transplantation. Zhao et al. recently conducted a meta-analysis comparing MSC therapy as induction therapy for kidney transplantation to traditional regimens. Their analysis, which included four trials with a total of 197 patients, revealed that MSC therapy resulted in a lower infection rate compared to traditional therapies within 1 year. However, no significant differences were observed between the two protocols regarding one-year acute rejection, delayed graft function, or graft function (Zhao et al., 2021). Nonetheless, the advantages of MSC therapy warrant further investigation through well-designed, multicenter randomized controlled trials (RCTs) with larger sample sizes and extended follow-up periods to assess its long-term efficacy and potential adverse effects.

## 7 Enhancement of transplanting cell function and optimization

One potential issue with the conventional intravenous (IV) administration of MSCs in clinical trials is their reduced efficacy, attributed to the cells’ inability to adapt and properly engraft within the target tissue. MSCs often undergo apoptosis due to the harsh conditions induced by tissue injury, including the presence of reactive oxygen species (ROS), ischemia, and anoikis (Song et al., 2010). The IV-administered MSCs frequently experience low engraftment rates as they tend to become sequestered in non-target organs such as the liver, lungs, and spleen (Sanchez-Diaz et al., 2021). Futhermore, the regenerative potential of MSCs heavily relies on their paracrine actions, which can be significantly impaired in CKD patients. Incase of AKI, the diagnosis of AKI often involves the measurement of rising creatinine levels, which can delay MSC administration and potentially lead to irreversible kidney damage (Silva et al., 2018; Zhao et al., 2019).

A novel *ex vivo* method, SBI-101, designed to simulate dialysis, has demonstrated increased efficiency in a Phase I/II trial (NCT03015623, Table 2) (Swaminathan et al., 2021). This method employs BMMSCs immobilized in the extracapillary space of a hollow fiber hemofiltration device, allowing for controlled and extended exposure of circulating blood to these cells (Figure 1). One limitation of this system is circuit clotting, necessitating the use of anticoagulants. Notably, the treatment exhibited anti-inflammatory effects, with reduced levels of TNFα and IFNγ pro-inflammatory markers and increased levels of anti-inflammatory markers such as IL-10 and TGF-β1 compared to the sham group. This early-stage study focuses on safety rather than clinical efficacy, with a small sample size (16 participants: 12 treatment and 4 sham controls). Preconditioning of MSCs presents a potential solution for enhancing its function. Melatonin, known for regulating circadian rhythms by maintaining low daytime and high nighttime blood levels, is a widely used preconditioning agent for MSCs in preclinical studies (Zhao et al., 2020). Melatonin possesses antioxidant and anti-inflammatory properties, providing protection to organs, including the kidneys, from dysfunction and supporting immune function. It may improve MSC therapy outcomes by mitigating oxidative stress and apoptosis through both receptor-dependent and independent pathways. Melatonin preconditioning has demonstrated improved therapeutic effects in various animal models and is considered safe with minimal side effects, though its efficacy in kidney disease remains uncertain (Zhao et al., 2020). Several preclinical studies, including one by Chen et al., have explored the impact of melatonin and ADMSCs) in rats with sepsis-induced AKI (Chen et al., 2014). The combination of melatonin and ADMSC treatment preserved kidney structure and function, reduced kidney damage, inflammation, and oxidative stress, and improved survival. This treatment significantly lowered markers of fibrosis, apoptosis, and DNA damage, especially when combined, and increased antioxidant protein levels, indicating protection against oxidative damage. Zhao et al. investigated the effects of melatonin-preconditioned human ADMSCs on human kidney proximal tubular cells (HK-2) in a Cisplatin-AKI model (Zhao et al., 2015). Melatonin enhanced ADMSC proliferation through MT1 and MT2 receptor activation, as confirmed by Western blotting, and involved P-Erk1/2 and P-Akt signaling pathways. Melatonin-treated ADMSCs (M-ADMSCs) significantly increased HK-2 cell proliferation and migration compared to untreated ADMSCs. In a cisplatin toxicity model, the conditioned medium from M-ADMSCs provided better protection against oxidative and apoptotic damage, associated with higher expression of prosurvival, antiapoptotic, and antioxidative proteins. The study by Mias et al. in rats with IRI AKI reached similar conclusions (Mias et al., 2008). Melatonin protected MSCs from apoptosis and enhanced the secretion of proangiogenic and mitogenic factors, thereby improving MSC survival and renal function. The effects of melatonin, mediated through MT1 and MT2 receptors, bolstered MSCs’ prosurvival properties and antioxidant defenses. Given its safety profile and ability to be removed before cell injection without loss of benefits, melatonin shows promise as a preconditioning agent for MSC-based therapies aimed at organ repair (Mias et al., 2008).

## 8 Optimal cell dose and route of delivery

The standardization of cell therapy dosing protocols is essential for improving the reproducibility and efficacy of these treatments. Current clinical trials for AKI and CKD reveal a lack of consensus regarding the optimal cell dose, with various studies employing different dosing strategies, ranging from hundreds of thousands to several billion cells. Identifying the optimal dose is crucial for enhancing therapeutic efficacy, minimizing side effects, and improving patient outcomes. Key considerations in determining the optimal dose include the calculation method, delivery technique, and frequency of administration. The method used to calculate the dose—whether based on body weight, kidney weight, or volume can significantly impact the precision and effectiveness of the therapy. For example, while many CKD trials use body weight or BMI for dosing, this approach may introduce inconsistencies due to variations in kidney size, especially in diabetic nephropathy. The REACT trials utilize allometric scaling based on kidney weight, derived from 3D volumetric imaging, which offers a more individualized approach. In REACT’s phase I and II studies, patients received two injections over 6 months, with a dose of 3 × 10^6^ cells per gram of estimated kidney weight (Stavas et al., 2024). This method accounts for individual anatomical variations and avoids inconsistencies associated with body weight-based dosing. In contrast, studies involving stromal vascular fraction, such as the ELIXCYTE trial, measure the dose in milliliters (mL) rather than cell count, with doses ranging from 8 mL to 40 mL, corresponding to cell counts from 6.4 × 10^7^ to 32.0 × 10^7^ cells. These SVF trials also highlight the challenge of patient-to-patient variability, particularly when cells are autologous and thus inherently variable in number. For instance, in an SVF study, the number of cells ranged from 31.7 × 10^6^ to 142.7 × 10^6^ (Carstens et al., 2023). This variability raises important questions about the reproducibility and scalability of cell therapy across different patient populations. The Elixcyte study found no significant relationship between the dose of adipose-derived stem cells (ADSCs) and improvement in eGFR, underscoring the need for larger clinical trials to validate these findings. This finding aligns with a meta-analysis by Papazova et al., which also reported no dose dependency in cell counts or product administrations (Papazova et al., 2015). This suggests that beyond a certain threshold, the number of administered cells may not significantly impact clinical outcomes, emphasizing the importance of the delivery method and the biological environment in which these cells operate.

The delivery method is also crucial in determining the effective dose. Studies indicate that localized delivery to the kidney cortex, as used in the REACT trials, may allow for a lower dose compared to systemic delivery, which is more susceptible to cell loss due to first-pass effects in the lungs and liver. Systemic delivery may require a second bolus dose to ensure that cells effectively reach the target organ, as injecting a second dose may help saturate certain receptors in the lungs and other non-target organs, thereby enhancing the likelihood of cells reaching the kidney (Fischer et al., 2009). Researchers continue to explore the optimal number of injections, with many trials investigating whether one or two injections is preferable. Additionally, the frequency of administration is critical; animal studies suggest that weekly MSC administration significantly improves kidney function compared to a single dose, highlighting the need to optimize both dose and frequency for effective ADSC treatment in CKD patients (Lee et al., 2010).

Translating of the cell doses from animal to human trials presents additional challenges. For instance, a trial using urine-derived stem cells for CKD treatment involves doses ranging between 1.0 × 10^8^ and 3.0 × 10^8^ cells, which is notably higher than the typical range of millions of cells used in other studies. Pre-clinical studies on rat models often use doses in the range of 10^5^ to 10^6^ cells, which are lower than those employed in human trials (Zhang et al., 2020). The rationale for such high dosages in human trials is unclear, as the trial is still in the recruitment phase with no available reports. A review of MSC use in AKI therapy indicates a significant mismatch between preclinical and clinical dosing data, with rodent studies typically using 50 million cells/kg, while human studies use about 1-2 million cells/kg (Sávio-Silva et al., 2020). This discrepancy may introduce a bias in clinical practice due to the lower dosages used in humans. Lastly, individual sensitivity to treatment must be considered. In a study of patients treated with REACT, 32% (7 patients) exhibited a positive eGFR slope post-injection, with 4 being “high responders” and 3 “moderate responders.” These responders showed an annualized eGFR improvement of 5.88 mL/min per 1.73 m^2^, compared to a decline of −3.96 mL/min per 1.73 m^2^ in “low responders.” The high/moderate responders had a slightly higher baseline eGFR (38.9 mL/min per 1.73 m^2^) and a lower urinary albumin-to-creatinine ratio (UACR) than the low responders. The cumulative dose of REACT was similar between groups. Additionally, a longitudinal model suggested that changes in eGFR might be influenced by RET expression in the REACT product, with a trend toward significance (P = 0.09) (Stavas et al., 2022).

Overall, due to the absence of standardized protocols in cell therapy studies, definitive conclusions on the optimal dosage remain elusive. Generally, multiple injections over a treatment period appear advisable for cumulative effects. Furthermore, tailoring dosages based on individual patient factors such as kidney size, disease severity, genetic markers, and treatment sensitivity could enhance therapeutic outcomes across diverse patient populations and disease stages.

A comprehensive review encompassing 469 preclinical and 50 clinical studies on MSC use for kidney diseases revealed that the majority of preclinical studies reported renoprotective effects of MSCs, irrespective of the delivery method (Kresse et al., 2023). This review categorized delivery routes into systemic, intravenous, and local injections into the target organ. In AKI models, both local and systemic delivery routes have shown comparable improvements in kidney function, though local delivery was favored in some studies. A meta-analysis indicated that systemic delivery had a slightly superior effect on reducing serum creatinine levels (Wanyan et al., 2023). Out of the reviewed clinical trials, 25 utilized systemic injection (either intravenous or intra-arterial infusion), three employed local delivery (renal artery or renal parenchyma injection), one used intramuscular delivery, and the remainder did not specify the method. Clinically, systemic delivery is preferred due to its less invasive nature compared to local methods. Additionally, Papazova et al., found that systemic IV-administration is the most well-supported by evidence and feasible for patients in preclinical studies (Papazova et al., 2015). Although less common, local MSC administration via the renal artery has proven feasible and safe in patients with renal vascular disease. The higher MSC doses used in preclinical studies compared to clinical ones suggest that local delivery may be advantageous for achieving therapeutic cell quantities in humans.

## 9 Conclusion and future perstectives

As demonstrated above, diverse cell therapies hold significant promise for treating AKI and CKD. Nevertheless, current research is hindered by several common limitations. First, the selection of patients at different CKD stages may introduce bias. Second, most studies administer treatments without considering the underlying etiology of the disease. Only a few trials, such as the REACT studies, specifically target diabetes-related cases. This is problematic because different etiologies can lead to distinct types of kidney damage, which may affect the efficacy of the treatment. Third, there remains no consensus on the optimal cell dosage. The absence of a standardized dosing protocol may lead to inconsistent results across studies, difficulties in conducting meta-analyses and systematic reviews, and challenges in clinical implementation. Finally, the small sample sizes in many studies contribute to a lack of randomization, low statistical power, limited generalizability, a higher risk of false positives, and potential biases.

## Abbreviations

AKI, Acute kidney injury; CKD, Chronic kidney disease; ESRD, End stage renal disease; GFR, Glomerular filtration rate; RRT, Renal replacement therapy; MSC, mesenchymal stem/stromal cell; BMMSC, Bone marrow-derived MSC; ADMSC, Adipose-derived MSC; iPSC, induced pluripotent stem cell; SSC, Spermatogonial Stem Cell; GPSC, Germline progenitor stem cell; PAC, Proangiogenic Cell; ECFC, Endothelial Colony Forming Cell; HUVEC, Human umbilical vein endothelial cell; ROS, Reactive oxygen species; IRI, Ischemia-reperfusion injury; TEC, Tubular epithelial cell; IV, Intravenous; EV, Extracellular vesicles; SVF, Stromal vascular fraction; REACT, Renal autologous cell therapy; SRC, Selected renal cell; EPC, Endothelial progenitor cell; AE, adverse event; MAE, minor adverse event; SAE, severe adverse event.
